# “I Missed My Other Oncologist”: Established Relationships as Barriers and Facilitators to Accessing CAR‐T and Autologous Transplantation

**DOI:** 10.1002/cam4.71425

**Published:** 2025-11-27

**Authors:** Zachary A. K. Frosch, Clark Meshack, Caitlin Meeker, Asya Varshavsky‐Yanovsky, Rashmi Khanal, Michael Bromberg, Ashwin Chandar, Emmanuel Quien, Jordan Carter, Shazia K. Nakhoda, Marcus Messmer, Charissa Montgomery, Jason A. Incorvati, Nadia D. Ali, Michael J. Styler, Carolyn Y. Fang

**Affiliations:** ^1^ Department of Hematology/Oncology Fox Chase Cancer Center Philadelphia Pennsylvania USA; ^2^ Cancer Prevention and Control Research Program Fox Chase Cancer Center Philadelphia Pennsylvania USA; ^3^ Department of Bone Marrow Transplant and Cellular Therapies Fox Chase Cancer Center/Temple University Hospital Philadelphia Pennsylvania USA; ^4^ Section of Hematology Temple University Hospital Philadelphia Pennsylvania USA; ^5^ Section of Hematology Thomas Jefferson University Philadelphia Pennsylvania USA; ^6^ Division of Hematology and Oncology University of Pennsylvania Philadelphia Pennsylvania USA

## Abstract

**Background:**

Autologous stem cell transplantation (ASCT) and chimeric antigen receptor T‐cell therapy (CAR‐T) can provide significant clinical benefit, but are not equally available to all patients. Oncologist continuity and trust are important to patients, and may act as barriers to receipt of therapy. However, detailed data are lacking on the mechanism by which these might affect receipt of therapy, or how this barrier may be overcome.

**Methods:**

We conducted a qualitative interview study with patients with non‐Hodgkin lymphoma (NHL), classic Hodgkin lymphoma (cHL) or multiple myeloma (MM) eligible for or treated with ASCT/CAR‐T. Participants were recruited from a multi‐site academic health system that includes a safety net hospital considered to be one of the most racially inclusive in the country. Interviews were independently coded by two trained coders.

**Results:**

Forty patients participated. Half were female, 65% had multiple myeloma, 45% identified as Black or African American, 22.5% had an income less than $30,000 and 33% were insured with Medicaid. In addition to treatment‐specific factors and logistical factors such as housing and transportation, patients identified the need to establish care and trust with a new provider as potential barriers to receipt of therapy. This process could be facilitated by the perception of strong communication and existing relationships between patients' established and new providers.

**Conclusions:**

In addition to logistical and clinical factors, several factors centering around existing and newly required patient‐provider relationships may influence patients' acceptance of ASCT/CAR‐T. Acceptance may be enhanced by addressing relationship‐based barriers in a manner that emphasizes continuity and helps build trust with a new provider, such as by explicitly demonstrating seamless communication among care teams.

## Graphical abstract

In addition to logistical and clinical factors, several factors centering around existing and newly required patient‐provider relationships may influence patients' acceptance of autologous transplant and CAR‐T. This may be enhanced by addressing relationship‐based barriers in a manner that emphasizes continuity and helps build trust with a new provider, such as by explicitly demonstrating seamless communication among care teams.

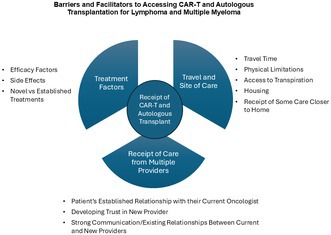

## 1 Introduction

Treatments such as autologous stem cell transplantation (ASCT) and chimeric antigen receptor T‐cell therapy (CAR‐T) can provide significant clinical benefit for patients with blood cancers [1, 2, 3, 4, 5, 6]. With indications for cellular therapy expanding, they are poised to play an increasingly prominent role in treatment. Yet studies suggest that not all patients who need these therapies can access them [7, 8]. Moreover, disparities exist in their use. Black and Hispanic patients, patients insured by Medicaid, patients living in lower‐income and less‐educated neighborhoods, and patients living further from a transplant center are less likely to receive these treatments [9, 10, 11, 12]. Of further concern, even as overall utilization of these therapies improves, disparities in access may be widening [13].

Multiple factors can interact to facilitate or hinder access to these treatments [14, 15, 16]. These can include clinical factors, insurance coverage [17], and those related to supportive services [18]. However, patient and provider factors centered around the clinical encounter [19] can also affect receipt of therapy. Given that ASCT and CAR‐T are only offered at specialized centers, many patients will need to receive therapy from a provider not already on their established care team. Prior research has shown that continuity with their existing cancer care provider is important to patients with blood cancers when they are considering treatment choices [20]. Longstanding relationships facilitate trust in providers [21], and continuity may be of even greater importance to some patients at higher risk for disparities in access [20, 22].

Despite the importance of these factors, less is known about the details of patients' decision‐making process when faced with trade‐offs inherent in therapy selection. In particular, it is not known what factors are driving patients' strong desire to maintain oncologist continuity, or how best to overcome these barriers and facilitate patients' trust in the multi‐team care often required for receipt of ASCT/CAR‐T. To address this, we conducted a qualitative study consisting of semi‐structured interviews with patients with non‐Hodgkin lymphoma (NHL), classic Hodgkin lymphoma (cHL) or multiple myeloma (MM) who were eligible for or treated with ASCT/CAR‐T. Our use of qualitative, semi‐structured interviews was designed to allow patients to elaborate on the mechanisms driving the associations seen in prior quantitative studies, and to uncover factors that may not be adequately captured by retrospective or survey data. The interviews were guided by the Health Equity Implementation Framework [19], which was developed to advance the uptake of new therapies while simultaneously reducing health disparities.

## 2 Methods

### 2.1 Eligibility and Setting

Eligible patients were diagnosed with NHL, cHL or MM and received or were considered eligible for ASCT or CAR‐T. Patients were recruited from a multi‐site academic health system that includes a safety net hospital considered to be one of the most racially inclusive in the country [23]. In this health system, patients may receive oncology care across multiple practice locations, but both ASCT and CAR‐T are performed at a single practice site. Patients who received prior, concurrent, or subsequent care outside the system were eligible.

### 2.2 Recruitment

Potential participants were identified from participating providers and the electronic health record. Permission to contact potential participants was obtained from the patients' established providers. Recruitment letters were mailed to participants, and up to three follow‐up phone calls were made to non‐responders. We purposively oversampled both non‐White and/or Hispanic respondents and those who received care at the safety net hospital, in order to capture the experiences of those most at risk for reduced access. Participants received a $25 gift card for participation. Our initial sample size estimate of 40 participants was based on the number of interviews typically required to reach thematic saturation [24]. This is often achieved after 12–16 interviews [25, 26], and can range from 20 to 40 interviews when exploring themes that cut across multiple subsamples [26]. Therefore, a conservative sample size estimate of 40 interviews was selected as the initial target for this study. Based on the iterative development of the coding dictionary as described below, thematic saturation was achieved with this sample size.

### 2.3 Data Collection

A 45‐min, one‐time semi‐structured interview was conducted with consenting participants between October 28, 2023 and April 3, 2024 either in person, by video conference, or by phone (based on participant preference). Format flexibility was provided to maximize input across a diverse population of participants with differing needs. Development of the interview guide was informed by the Health Equity Implementation (HEI) Framework [19], a review of the literature on disparities in access to cellular therapies, and the investigators' preliminary data [20]. Participants consented to interview recording, and recordings were subsequently transcribed and de‐identified.

### 2.4 Analysis

Interview transcripts were coded independently by two trained coders (CMe & CMe) using modified grounded theory with NVivo software (Version 1.7.1, QSR International, Burlington MA) [27]. Inter‐rater reliability was measured using kappa statistics in NVivo with high reliability being defined as > 0.8 [28]. Interview transcripts were coded both based on emergent codes from the interview data and a priori codes based on domains of the HEI framework which informed the interview guide. Coding proceeded iteratively. The analysis team met at least weekly to review reliability and a third coder (ZAKF) was employed in areas where inter‐rater reliability between the two primary coders was < 0.8. The codebook was refined and re‐applied to the interview data iteratively until high inter‐rater reliability was achieved (kappa > 0.8). Then, the final coding dictionary was applied across all transcripts. Coded transcripts were then used to produce thematic reports summarizing study findings.

The study was approved by the Institutional Review Board at Fox Chase Cancer Center (#22–8006) and all patients provided informed consent.

## 3 Results

### 3.1 Participant Characteristics

To reach our target sample size of 40 participants, we contacted 66 potential participants. Forty‐five consented, five withdrew prior to the interview, and 40 patients completed the interview. Of all contacted patients, 61% completed the interview. Half of the participants were female, 18 (45%) identified as Black or African American, 10 (25%) had a high school education or less, 14 (35%) had an income below $50,000 and 9 (22.5%) less than $30,000. One‐third (13, 33%) were insured with Medicaid or were dual eligible. The majority (26, 65%) had multiple myeloma, 9 (22%) had non‐Hodgkin lymphoma, and 5 (12.5%) had Hodgkin lymphoma. Almost half (17, 42.5%) were initially seen for their cancer outside of the health system (Table 1).

**TABLE 1:** Participant characteristics.

	*n* (%)
	*n* = 40
Age	
18–49	4 (10)
50–64	15 (37.5)
65+	21 (52.5)
Gender	
Female	20 (50)
Male	20 (50)
Race	
Black/African American	18 (45)
White	21 (52.5)
Other	1 (2.5)
Ethnicity	
Hispanic/Latinx	1 (2.5)
Not Hispanic/Latinx	39 (97.5)
Education	
High school or less	10 (25)
Some college	18 (45)
Bachelor's degree	5 (12.5)
Master's degree	4 (10)
Trade school	1 (2.5)
Prefer not to say	2 (5)
Income	
Less than $30,000	9 (22.5)
$30,000 to $50,000	5 (12.5)
$50,000 to $75,000	6 (15)
$75,000 to $100,000	4 (10)
More than $100,000	8 (20)
Prefer not to say	8 (20)
Insurance	
Commercial	12 (30)
Medicaid	13 (32.5)
Medicare	10 (25)
Managed medicare	4 (10)
Dual eligible	1 (2.5)
Cancer type	
cHL	5 (12.5)
MM	26 (65)
NHL	9 (22.5)
Initial site of care	
Outside the health system	17 (42.5)
Academic practice within the health system	15 (37.5)
Bone marrow transplant/CAR‐T practice within the health system	1 (2.5)
Safety net hospital practice within in the health system	7 (17.5)
Receipt of ASCT/CAR‐T	
ASCT	34 (85%)
CAR‐T	3 (7.5%)
Declined	3 (7.5%)

### 3.2 Thematic Summary

Several themes emerged from the interview data regarding receipt of recommended therapy. In the first two themes, participants focused on more concrete areas such as those related to treatment‐specific factors, travel, or other logistical issues. However, participants also identified a number of interpersonal factors that can affect receipt of treatment (Theme 3), including those related to oncologist continuity and establishing trust with new providers.

### 3.3 Theme 1: Treatment Factors Affecting Patients' Preferences

Participants identified multiple treatment factors that affected their therapy preferences (Table 2). These factors included not only general efficacy, but also either the potential for cure (patients with lymphoma) or for a treatment‐free interval (patients with MM) (Table 2). Patients balanced efficacy against short‐ and long‐term side effects. They considered the need for hospitalization, though flexibility in the timing of transplant potentially mitigated this for some. When discussing side effects, patients with MM focused more on short‐term side effects and quality of life, while those with cHL/NHL often mentioned long‐term side effects (Table 2).

**TABLE 2:** Theme 1: Treatment factors affecting patients' preferences.

Sub‐themes	Illustrative quote(s)
*Facilitators*	
Efficacy	“I factor in how well the treatment's going to work. Not necessarily what I'm going to have to go through with the treatment but the outcome of the treatment.” (52, NHL) “Obviously the most effective one is what I would be interested in” (58, NHL) “If I have a chance to survive a little longer, maybe I should try this.” (76, MM) “I just was pretty much wanted them to be as aggressive as possible to get it under control, so I believe the stem cell transplant did exactly that.” (381, MM)
Curative potential	“the goal was to cure. That's what I was focusing on and I didn't want to maintain or live comfortably, I want to get rid of it.” (728, NHL) “it seemed like that had to be the way I had to go if I wanted a chance at living longer and maybe a chance of even being cured” (72, NHL) “the numbers basically were significant for people getting cured going that route. So, it was the best choice” (50, cHL)
Potential for a treatment free interval	“I could just keep going through different courses of medication but if I went for the bone marrow transplant it seems like the better option to get it reduced faster so that I would be on less medication. So this is the reason that I chose to have the bone marrow transplant…because I knew I was probably going to have to be on medication for the rest of my life because there is no cure for multiple myeloma.” (435, MM) “Chemotherapy was the only other than bone marrow transplant…I understood that in order to go that route that I would be on chemotherapy for the rest of my life.” (375, MM) “What you really want to accomplish is the least medication…I don't want to be hooked up to a machine all the time” (435, MM)
Flexibility in treatment timing	“The only choice I had was when they took my stem cells, whether I was going to do it put back in right away or wait and have them frozen and I chose to wait only because I'm working and I can only take off in the summer time, so we would wait until then and [my doctor] was okay with that.” (649, MM) “it was better for me to have the stem cell transplant I felt in the summer when there's less risk of infection and my kids aren't in school and stuff like that.” (58, CHL)
Recommendation of provider	“I gave [my doctor] full autonomy. Whatever you suggest, I'll do.” (340, NHL) “I went with what my doctor recommended.” (781, MM)
*Barriers*	
Short term side effects	“I just wanted to be assured that I would not have a poor quality of life.” (375, MM) “I just felt that the bone marrow transplant is such a drastic move initially. In the long run, it's good, but initially it's draining. It's not something I would rush into, let's put it that way unless [it were] absolutely necessary.” (375, MM) “I went for the chemo, because there was going to be too many side effects [with a stem cell transplant] and I didn't want to do that particular option…I didn't feel that maybe my body could handle the three weeks [in the] the hospital, and isolation and just going through all the side effects that might occur during that treatment.” (130, MM)
Long term toxicities	“Important factors definitely, how is it going to affect me in the long run? Like I said as a younger patient, some of these treatments have side effects that can lead to serious problems…we can handle short‐term side effects. They stink but those aren't nearly as important as I really look at the long term side effects. Is this going to lead to a problem for me down the road…” (88, cHL) “Just about what was gonna work the fastest and that was still gonna have the minimum effects in the long term for me. That was like one of my main concerns.” (439, cHL)
Hospitalization	“I'm trying to avoid the hospitalization… I was there with a stem cell transplant two weeks, you know each time. And then of course I couldn't do anything for 100 days which was fine. But in this case I tried to avoid the hospitalization, so I chose this one over…[the treatment that] would require hospitalization” (71, MM) “my husband was home sick. And I couldn't leave him alone, I'd have to have somebody here with him while I went to the hospital.” (142, MM)
*Other facilitators and barriers*	
Novelty of treatment	“this CAR T thing is coming and I want to be at the hospital that's going to be doing the latest and greatest.” (30, MM) [*Facilitator]* “[my doctor] was up to [date on]…all the most recent…technologies, surgeries, options and I was so impressed.” (52, NHL) [*Facilitator*] “I was very feisty. I had my guard up, yes, I did and I didn't want to hear nothing about being a guinea pig and none of that stuff. Because that's what research is about. Research is about trying to find out what makes that person tic and what makes that person not tic and stuff. That's what research is about, it's about being a Guinea pig.” (197, MM) [*Barrier*]
Anecdotal connections with other patients	“I was an internet person, and from what I read on my own about the stem cell, I've seen a lot of people that had success rates from it and like I did my homework, I looked it up, I read about other people that moved on with their life after they had the treatment and I was 100% for it.” (52, NHL) [*Facilitator]* “Yeah. And I had my husband's cousin had a friend who went through the bone marrow transplant, I think three months ahead of me. So I got to talk to her as well and it makes you feel better because it gives you some information and any questions that you had. They would answer these questions for me and it just gives you a little more stability and where you're going to be going.” (435, MM) [*Facilitator*] “Yes, because another woman I knew had the cell transplant and she ended up passing.” (137, MM) *[Barrier]* “Before we did the CAR‐T, there was a Facebook group for mantle cell lymphoma and there was a lot of discussion about different treatments and what they had done to people. … when certain people start telling you over and over and over again certain things are not what they would have done, you get the idea that's not something for you.” (102, NHL)

Novelty of the therapy acted as a facilitator for some and a barrier for others. Some participants described preferring “cutting‐edge” therapies while others preferred those with more established track records.

My decision was based on researching the doctors and cutting‐edge technology. (619, MM)

I mean I don't know that…I'd want to take that chance on top of everything else that was going on. I understand things are newer and you have to try things. It would depend… (728, NHL)

Participants reported that the recommendations of others factored into their choices. They frequently mentioned that their provider's recommendations carried significant weight in their treatment choice.

When you have something like this you have to rely on what the doctors tell you, because they're the experts. (58, NHL)

I would rely on my physicians. (777, MM)

In addition to their provider's recommendation, participants also considered the positive and negative experiences of others they knew either directly or indirectly (Table 2).

### 3.4 Theme 2: Travel and Site of Care

Participants identified several factors related to travel and where they received their care that affected their treatment choices. Patients viewed longer travel as a barrier to receipt of treatment.

So I thought that was a big factor. Driving 15‐20 minutes compared to an hour. I thought that was a lot of time saving. (130, MM)

We just picked the one that we thought would be the best distance for us because in case my husband had to go back and forth. (435, MM)

Some participants reported a willingness to travel for what they perceived as high quality care or treatments, or to see an existing provider.

I was getting good care and I prefer to drive further than not to get good care. (30, MM)

I think if it's explained to the patient that at this facility they have this expertise whereas we don't have this expertise or we don't have the particular treatment…and that you need to go to another facility then that's what you have to do. (421, MM)

My one doctor moved to another office and sometimes I do travel further to get to him because I want to stay with him. (435, MM)

Other participants were more skeptical that they needed to travel for specialized care.

So they might be doing the experimental stuff and they might be doing the cutting edge stuff…but you can go to any oncologists these days and get a bag of IV that's full of the chemical you need. (347, NHL)

Participants identified several barriers to travel, including housing, transportation, and physical limitations due to treatment or other co‐morbidities (Table 3). They also identified several factors that may overcome these barriers, including local housing options provided either by family or their care team and transportation assistance.

**TABLE 3:** Theme 2: Travel and site of care.

Sub‐themes	Illustrative quote(s)
Physical limitations as a barrier to travel	“after that treatment I was always pretty tired and so being able to get home and just get back to bed, I don't think I could have made a four and half, four‐hour drive after treatment. So I did factor in the distance.” (347, NHL) “I think it factors greatly because of the distance. Having the stenosis in my back it was very painful for me to drive up there.” (375, MM)
Satellite offices/receipt of some care closer to home	“if they were able to be closer to home for follow‐ups and stuff, that'd be great. Like if there's a satellite office.” (781, MM) “the number one complaint is that people have to drive so far. So if there were different satellite sites, that would be great.” (375, MM)
Local housing	“I would have moved…if I had to, to get my treatment there… Then when I did my stem cell transplant [I stayed] at the transplant house and they only charged me like $35 or $40 a day and I lived up there for three months.” (347, NHL) “My daughter lives 15 minutes…It's one of the reasons I chose [to be treated there]” (375, MM) “It takes me two hours to get up there, but once I'm there then I get my treatment and then I go to my daughter's and stay there.” (375, MM)
Access to transportation	“I only have to catch one bus, but in the beginning, my friends would pick me up and go with me and bring me back home, and then the Leukemia Society told me that they had a grant for $500 if I needed somebody to take me back and forth to the doctors that would pay for the gas.” (137, MM) “We was concerned about the transportation with the gas and all that but it got taken care of, there wasn't no issue no more.” (197, MM) “I had to take a train and then walk to the hospital. [it took me] basically the whole day.” (50, cHL)

### 3.5 Theme 3: Receipt of Care From Multiple Providers

Participants differed in their attitudes towards seeing multiple providers as part of their cancer treatment, and identified a number of barriers related to this factor (Table 4). Participants frequently discussed their strong relationship with their existing oncologist as a barrier.

I have built a rapport with that doctor, so I would just rather stay with him. (643, MM)

**TABLE 4:** Theme 3: Facilitators and barriers to seeing multiple providers.

Sub‐themes	Illustrative quote(s)
*Barriers to seeing a new provider*	
Relationship with current oncologist	“I've established a trust with that particular doctor. It's not only being trusted with their medical skills but also relationship. That doctor gets to know how I operate and my personality to a certain level and I get to know how that doctor operates and either whether or not I accepted that. I don't need to have two or three doctors.” (684, MM) “I missed my other oncologist…” (58, NHL)
Concerns about missing important pieces of the clinical course to date	“And all I could think of was that, and this might not be fair to anybody, but this is what I thought, what if you skipped over something? What if you missed something, how would I know? How would you know?” (142, MM) “I feel like with my original doctor, that we went on this journey together and they remember all the bumps and all the different things that we talked through and strategized. It's hard to communicate all that to a new doctor that hasn't been with you for the whole time. You have to tell them all those twists and turns that you went through and I'm sure it's difficult for them to memorize this whole story.” (88, cHL)
Different communication/care styles between providers and teams	“The hardest thing about that was dealing with the different personalities…” (619, MM) “It was like a whole new [inaudible] other people to get used to. Other different nurses and that's all. It wasn't hard. It's all how you look at it, I think I could have made it harder on myself, but what was good would that have done me?” (58, NHL)
Perception that care currently going well	“I wouldn't want to do that…I would like to stick with who I have, because they are the ones that got me in remission now.” (229, MM) “I feel like if I've been helped by these doctors I'm not going to change my doctors.” (435, MM)
Employment/Financial	“I had to take time off work and do this initial consultation before I could get my treatment and that was a little frustrating. Actually, it was a lot frustrating because I also had medical bills for said initial consultation and when you're fighting cancer you have enough bills as it is…” (347, NHL) “Everything works good. I'm not working, so it's no problem. I might go back to work, I'm not sure, because I'm not sure I want to handle that again.” (387, MM)
Information overload at the beginning	“…because a person is usually in a fog hearing so much information.” (684, MM) “…because it's just a lot to take in.” (728, NHL)
Initial skepticism	“So I mean it's hard to build up trust in a new doctor because you never really, it's like being in a war with somebody until you're fighting alongside somebody, you don't know what they expect.” (52, NHL) “Well, because my father always says don't trust anybody, or trust the person but not the demons in them…I might be a little snarky or sarcastic in the beginning, but then once the rapport, the good starts. Yeah, it's all about communication, not talking to a robot.” (340, NHL)
*Facilitators of seeing a new provider*	
Perception of strong communication between providers	“I was comfortable and probably because over here it was her friend and colleague and I just felt a little special.” (76, MM) “No difficulty, because they will keep in touch with each other. What information this doctor does, he fax it down to that doctor and this doctor down there fax it to back up to him.” (387, MM)
Existing relationships between providers	“I think my doctors worked well together and I think there—because they had a previous—they worked together before, it was easy.” (381, MM) “I had no issues with it because apparently they worked together as a team and my original oncologist was confident enough to recommend me to the BMT doctor.” (637, NHL)
Belief that new provider was prepared for initial meeting	“What I was impressed with is that when I saw [doctor], he seemed very familiar with my case…So I felt he had reviewed my history very well before meeting with me.” (30, MM) “Everyone knew where I was at in my treatment, it wasn't much that I had to explain to them. It seemed like all the doctors would speak to each other before—…It's like they already knew who I was.” (439, cHL)
Appreciation that providers play multiple roles in care and may specialize	“They were all involved with the treatment. It wasn't like I got like a second or third opinion now. It was that [there were] different doctors for the different types of treatment I received.” (421, MM) “It's just that right now I have to see her, because she's my doctor right now because of the type of treatment that I needed.” (58, NHL)
Time spent with patient	“The amount of time that he spends with me when I'm there for a visit or the time on the phone. I don't feel rushed. I feel that I can ask a question or questions regardless of how many I have and he answers them and I think that's an important thing is that I don't feel rushed.” (375, MM) “You don't want to feel rushed and you want to feel like he takes his time, he listens to what you're saying.” (435, MM)
Provider's demonstration of knowledge, including disease specific	“Knowledge of the specific disease that you have like I know I have multiple myeloma and the knowledge that the doctor presents of her knowing the disease, the side effects of the chemo, how the chemo is going to affect you, and just basic knowledge of how to treat this disease and how to bring it in remission is what makes you trust what the doctor says a whole lot sooner.” (619, MM) “I trusted him right away. He seemed to know what's going on. He was going through some different stats about different things” (71, MM)
Provider's confidence	“The way he explained things to me in a way he handled himself and how confident he seemed when he told me the different options.” (72, NHL) “Like I was confident the way she was talking, she looked like—she sounded like she knew what she was doing” (50, cHL)
Perception of strong, individualized communication and empathy	“I guess I never felt alone, you know? I always felt like there was someone there who listened and that's a good step for trusting someone.” (71, MM) “They were honest and they were empathetic.” (347, NHL) “You listen to what they say and you know, I'd like to ask them questions of the doctor and see how they answer me. I like to, after you talk back and forth and you see how that doctor is treating you, you don't want to feel like he's talking so much and it's over your head or whatever.” (435, MM)
Recommendations of existing providers	“I didn't really consider anything else I think, because my regular oncologist had already recommended [location]…This bone marrow treatment area was where I needed to go, then when I got there, I felt comfortable with what they were telling me.” (58, NHL)
Other individuals' prior experiences with the practice	“I, again, went on the hearsay of other people as to the doctor that I chose.” (375, MM) “I just immediately came to trust them and people that I know have gone there for breast cancer and lung cancer, so immediately, I know I was in good hands.” (32, cHL)
Results of personal research on provider	“I did research on her and everything I read was really, really good.” (619, MM) “We are people that don't just pick any doctor we are people that do the research find the best doctor and then go to that doctor quite frankly no matter where they are.” (102, NHL)
Reputation of practice or institution	“He was at [location] and so I said, ‘Oh well, that makes me feel confident.’” (142, MM) “I did not do research on [doctor] because he's at [location] and I trust [location], you know what I mean?” (115, MM)
*Length of time needed to trust a new provider*	
Immediately	“For me no it's just one [appointment].” (347, NHL) “I would say initial impression it's very key. I guess first impressions they are lasting and that's it, first impressions.” (643, MM)
Few appointments	“So I think probably a couple appointments just so I really had my head wrapped around what was about to happen and got to know them a little bit better.” (58, NHL) “I would say a couple of appointments. I don't trust anyone right on the spot. You have to build trust and I think it takes a little bit of time to do that.” (375, MM) “It takes more than one appointment. It takes multiple appointments to build trust. It may take a month. It may take two weeks. It depends on the communication that you receive from your doctor” (619, MM)
Longer	“So finding somebody I was comfortable with was a priority with me. Shopping for a doctor, I wasn't uncomfortable doing that.” (643, MM) “It takes a few appointments. I would say 3–6 months.” (781, MM)

Participants discussed concerns related to new providers missing important pieces of their past clinical course in the transition, and that different communication and/or care styles between teams could also be challenging (Table 4). A perception that their care was currently going well was also noted as a barrier, particularly for patients with MM.

No I am fine with the one I have…because they tell me everything is good every time I go there. (620, MM)

Patients also identified potential employment‐related or financial concerns that might act as a barrier to receipt of multi‐team care (Table 4).

Participants identified several facilitators that might help enhance their willingness to see multiple oncologists. Patients' perceptions of strong communication between providers were frequently mentioned.

[initial physician] was always in constant contact with [transplant physician]…So I always felt like she was there, she was always saying what was going on with me and she was aware, so you never felt like you're abandoned passed on to the next person. (52, NHL)

Relatedly, participants also cited existing relationships between the different providers, as well as a belief that their new provider was prepared for the initial meeting (Table 4). Some participants expressed an understanding that providers may play specialized roles in their care, which facilitated their acceptance of multi‐team care (Table 4).

When establishing care with a new provider, participants reported needing varied amounts of time to establish trust. Some reported trust immediately on establishing care, others a few appointments, others even longer (Table 4). In contrast to time‐based intervals, some participants reported milestone‐based intervals tied to the receipt of therapy.

No, after my treatment, my BMT one, I trusted them. (217, NHL)

Some participants reported that they felt they had no choice but to trust their provider given the situation they were in.

This is my life that they're dealing with. So I have to trust them. (606, MM)

I trusted right away. I didn't fight it. I wasn't going to fight, I wanted to fight cancer. I didn't want to fight my doctor. (381, MM)

Participants reported that multiple factors facilitated more rapid trust in new providers (Table 4). One frequently mentioned factor was the amount of time a provider spent with the patient. Other factors included the provider demonstrating a high level of knowledge, including about the specific disease being treated, a provider's confidence, and the patient's feeling that there was strong and individualized communication accompanied by empathy. The recommendation of participants' existing care providers carried weight and facilitated more rapid trust in new providers. Relatedly, trust could be facilitated if individuals known to the patient had prior positive experiences with the provider or practice, or if a patient's personal research on a provider was reassuring. Finally, the perception of a positive reputation of the practice facilitated trust. Barriers to trust included being initially overwhelmed by the amount of information to incorporate, or an individual's predisposition to skepticism about what to expect (Table 4).

## 4 Discussion

In addition to logistical and clinical factors, our findings suggest that several factors centering around existing or newly required patient‐provider relationships may influence patients' acceptance of ASCT/CAR‐T. Willingness to travel further for treatment was influenced both by practical considerations (such as housing, transportation, and physical limitations), and by patients' beliefs about the degree to which care can or cannot be delivered locally. One key interpersonal barrier to being treated by multiple care teams was patients' strong relationship with their current oncologist. However, this may be overcome by patients' perception of existing relationships and strong communication between providers, both before and after the initial visit. The length of time patients reported needing to develop trust in a new provider varied from a single successful visit to after successful receipt of therapy. Both indirect factors (e.g., recommendations of existing providers), and factors directly related to the clinician‐patient interaction (e.g., time spent with the patient), facilitated more rapid trust. Our findings suggest that, in addition to managing logistical and practical considerations, patient acceptance of ASCT/CAR‐T may be enhanced by addressing relationship‐based barriers such as by explicitly demonstrating seamless communication among care teams.

In addition to addressing the clinical and logistical barriers that may potentially facilitate access to CAR‐T, patients reported that factors related to continuity with their established oncologist and establishing care with a new provider were important aspects of their care. In our prior quantitative work, participants reported that provider continuity was a valuable factor when selecting treatments [20]. The present study both validates and expands on these prior findings. Here, patients both confirmed that their relationship with their existing provider is an important factor in their therapy selection and expanded on specific aspects underlying this preference. Importantly, they identified how existing relationships and strong communication between different oncologists on their care team can increase their willingness to see multiple providers as part of their care. Given that the perception of strong collaboration appears important to patients, ensuring patients are aware of the conversations that frequently occur between collaborative oncologists may increase their comfort with multi‐team care.

Given the importance of continuity to patients, one potential facilitator that may help patients overcome the initial barrier of establishing care with a new provider is a collaborative telehealth visit. To date, the literature surrounding telemedicine use in transplant and cellular therapy largely centers on peri‐transplant and follow‐up care [29, 30, 31, 32]. Other studies have shown that the use of telehealth for specialized post‐transplant care may expand the geographic availability of these services. In one study, a graft vs. host disease (GVHD) clinic that offered both an in‐person and a telehealth option provided services via telehealth to a significant number of patients living over 100 miles from the clinic across multiple states [33]. Despite successes such as these, less is known about the use of telehealth in an initial collaborative visit with established and new providers. However, based on existing data and data from this study, collaborative telehealth visits may help both lower logistical barriers to establishing care while simultaneously demonstrating collaboration between primary oncology and transplant teams in real‐time. Its use in the initial visit setting should be further investigated.

The time needed to build trust with a new provider may also act as a barrier to receipt of therapy for some. Patients reported a wide range of time necessary to establish trust with a new provider. Some established trust within a first visit; others did not feel they had established trust until after they received therapy. Participants did suggest a number of factors that might facilitate more rapid trust, including the amount of time spent with the patient, the level of confidence and knowledge the provider projected, and the perception of strong communication. Given the time demands on oncologists, both strategies that free up time to spend with patients through increased efficiencies elsewhere (e.g., reduction in electronic medical record engagement burden) and that increase patients' perception of time spent [34] may be beneficial in facilitating trust in new providers.

Participants reported some clinical factors that can facilitate or hinder their receipt of ASCT or CAR‐T that were present across disease types, and others that were disease or situation specific. Consistent with prior work, patients valued treatment efficacy and tolerability [20], however the most frequently mentioned aspects of this differed depending on a patient's disease. Patients with lymphoma focused on the potential for cure and on long‐term toxicities, while those with myeloma focused on the potential for a treatment‐free interval and near‐term toxicities. Prior work has shown that, similar to patients with advanced solid tumors [35], patients with hematologic malignancies may not have an accurate understanding of their potential for cure [36, 37]. However, while not directly assessed in our study, many respondents reported important aspects of their decision‐making that were concordant with their disease characteristics (e.g., curability). Regardless of potential for cure, patients with both lymphoma and myeloma reported that treatment timing can be a factor. While potentially important to all patients, flexibility in the timing of treatment may be more easily addressed for some patients than others given common clinical situations in which these therapies are considered. To the degree possible, a focus on the above disease‐specific and treatment‐specific outcome factors to address any related concerns may facilitate receipt of therapy.

Participants also reported logistical factors such as travel and housing were important aspects to be addressed. While these factors have been reported in prior studies [18, 20], participants in our study specifically discussed the receipt of some care at satellite offices closer to home as a facilitator. This provides support for our prior quantitative finding that local follow‐up care may lessen the negative impact of increased travel time [20]. Multisite cancer care is increasingly common [38], and many large centers have satellite sites that can potentially be leveraged to facilitate access to specialized therapies [39]. Other studies have shown that multi‐site, complex care for patients with hematologic malignancies is potentially feasible. A shared care program for hematopoietic stem cell transplant recipients in which participants received approximately half of their post‐transplant care locally demonstrated similar clinical outcomes and improved early (day 100) post‐transplant quality of life for those receiving shared care with a local team [40]. Access implications were not the focus of that study, and further research around whether such models also address the barriers identified in our study and improve access to these therapies should be considered.

Our study has limitations. First, this is a qualitative study. Therefore, the degree to which each factor contributes to or hinders receipt of these treatments cannot be quantitated. However, our methods allow for patients to identify areas that were underexplored in prior quantitative studies, to elaborate on the mechanism behind factors that are difficult to quantitatively measure, and to offer potential solutions to identified barriers. Therefore, these data complement and expand on existing studies. Additionally, selection bias is a potential concern. However, our sample size is in line with or exceeds that of other qualitative studies in this area [41, 42, 43]. Moreover, our goal in this study was to hear from patients with diverse experiences, including those at risk of reduced access to these treatments. Therefore, we oversampled certain populations, resulting in a study sample that reflected diversity across race, education, and socioeconomic status.

## 5 Conclusions

In conclusion, patients reported that in addition to clinical and logistical barriers, addressing barriers related to oncologist continuity and the need to build trust with a new provider may facilitate access to therapy. Given that receipt of care from multiple providers is often required for ASCT/CAR‐T, the potential means to overcome these barriers that were identified by our participants should be further explored.

## Author Contributions

**Zachary A. K. Frosch:** conceptualization (lead), formal analysis (equal), funding acquisition (lead), investigation (lead), methodology (equal), writing – original draft (lead), writing – review and editing (equal). **Clark Meshack:** data curation (equal), formal analysis (equal), investigation (equal), project administration (equal), software (equal), writing – original draft (supporting), writing – review and editing (equal). **Caitlin Meeker:** data curation (equal), formal analysis (equal), investigation (equal), project administration (equal), software (equal), writing – original draft (supporting), writing – review and editing (equal). **Asya Varshavsky‐Yanovsky:** investigation (equal), resources (equal), writing – review and editing (equal). **Rashmi Khanal:** investigation (equal), resources (equal), writing – review and editing (equal). **Michael Bromberg:** investigation (equal), resources (equal), writing – review and editing (equal). **Ashwin Chandar:** investigation (equal), resources (equal), writing – review and editing (equal). **Emmanuel Quien:** investigation (equal), resources (equal), writing – review and editing (equal). **Jordan Carter:** conceptualization (supporting), funding acquisition (supporting), investigation (equal), resources (equal), writing – review and editing (equal). **Shazia K. Nakhoda:** investigation (equal), resources (equal), writing – review and editing (equal). **Marcus Messmer:** investigation (equal), resources (equal), writing – review and editing (equal). **Charissa Montgomery:** investigation (equal), resources (equal), writing – review and editing (equal). **Jason A. Incorvati:** investigation (equal), resources (equal), writing – review and editing (equal). **Nadia D. Ali:** conceptualization (supporting), funding acquisition (supporting), investigation (equal), resources (equal), writing – review and editing (equal). **Michael J. Styler:** conceptualization (supporting), funding acquisition (supporting), investigation (equal), resources (equal), writing – original draft (equal). **Carolyn Y. Fang:** conceptualization (equal), formal analysis (equal), funding acquisition (supporting), investigation (equal), methodology (equal), resources (equal), supervision (lead), writing – original draft (supporting), writing – review and editing (equal).

## Funding

This work was supported by NCCN Foundation.

## Conflicts of Interest

Dr. Zachary A. K. Frosch reports Advisory Board Fees from Seagen and research funding to the institution from Roche, Sanofi, Antegene, AstraZeneca, Genmab, AbbVie, Acerta, BeiGene, Merck and Genentech. Dr. Shazia K. Nakhoda reports honoraria and advisory board fees from Astra Zeneca and BTG/SERB and advisory board fees from Beigene.

## Data Availability

Data are not available due to privacy/regulatory restrictions.
